# Importance of Health Claims in the Adoption of New Breakfast Cereal Products in the UK

**DOI:** 10.3390/nu11123076

**Published:** 2019-12-17

**Authors:** Montserrat Costa-Font, Cesar Revoredo-Giha

**Affiliations:** Rural Economy, Environment and Society Department, Scotland’s Rural College (SRUC), King’s Buildings, West Mains Road, Edinburgh EH9 3JG, UK; Cesar.Revoredo@sruc.ac.uk

**Keywords:** health claims, new product development, nutrition claims, market success

## Abstract

Regular breakfast consumption has the potential to prevent the prevalence of NCDs and to improve the nutritional profile of diets. Given consumers’ interest in improving their diets, food suppliers are interested in introducing new cereal products making different health claims to capture consumers’ attention. The purpose of this study is threefold: first, it aims to understand whether UK food suppliers are working to increase the availability of breakfast cereals with healthy and nutritious attributes; second, it explores which companies are leading the launch of these products; and third, it assesses to what extent health and nutrition claims made by breakfast cereals have an impact on their market success. The study employs an assembled database combining data from Mintel Global New Products Database (GNPD) and Kantar Worldpanel Dataset (KWDS) for the UK. A hazard-based duration model was used to analyse the success of the new products launched in the UK market in 2011 following them up to 2015. Our results reveal that UK suppliers broadened the number of breakfast cereals on offer in the period 2000 to 2018, with a particular focus on multigrain cereals, porridge and granola. Health and nutrition claims were added to 27% of these products. Although consumers welcome healthy alternatives such as muesli, the impact of positional claims on the success of newly developed breakfast cereals is claim-specific. No clear pattern regarding the impact of health and nutrition claims is identified. However, other elements such as celiac-friendly ingredients and UK origin do have a positive impact on the success of breakfast cereals.

## 1. Introduction

Diet quality has been reported to be an important risk factor for non-communicative chronic diseases (e.g., diabetes, stroke, cancer) [1,2]. Poor-quality diets are associated with an inadequate intake of key micronutrients, as well as a lack of consumption of whole grains, fruit, nuts, seeds and omega-3 fatty acids, among other elements [1]. Micronutrient deficiencies such as in iron, vitamin A, D, magnesium and zinc, among others, are common in many developing countries and named as “hidden hunger”. An important step towards achieving a healthy and rich diet is a regular consumption of breakfast, which provides a higher percentage of micronutrients than other meals [3,4,5,6]. In 1960, Adelle Davis stressed the status of breakfast as the most important meal of the day [7] and later studies detailed that ideally between 15% and 25% of our daily energy intake should be consumed at breakfast [3,7,8].

Gaal [9] and Reeves [10] revealed that the majority of the UK population (around 95%) are regular breakfast consumers. Breakfast cereals such as porridge and muesli are also reported to contribute to more than 50% of the breakfast energy intake for the UK population [9,10,11,12]. When consumed regularly, breakfast cereals have been linked to an increase in the nutritional profile of diets, only with the exception of a high level of simple sugars associated with those ranges with a high added sugars content [13,14]. Furthermore, evidence suggests an inverse association between breakfast cereal consumption and body weight [15,16]. Paradoxically, despite the interest of consumers in healthy products and their health effects, the trend of breakfast cereal consumption in the UK exhibited a 4% decline in the volume of sales from 2014 to 2019 [17]. The reduction in breakfast cereals sales and consumption has been related to social and governmental pressure to reduce sugar, negative media coverage regarding ultra-processed foods, and changing breakfast habits with movement towards more convenient, healthy and on-the-go food products [17].

In terms of firm competition, the breakfast cereal market displays common characteristics with fast-moving consumer goods, which compete intensively to introduce new products [18]. Raubitschek described this kind of performance [19] as a model of product proliferation. A group of companies compete to introduce new products into the market, making health benefits and other types of claims, “hoping” that by doing so they will hit the jackpot, i.e., new products introduced into the market will become successful because they are taken up by consumers and remain on retailers’ shelves for a long time [19]. However, not all new products survive and between 60% and 80% of these new products eventually disappear from the shelves [20,21].

New breakfast cereals are introduced following consideration of several positional claims trying to attract consumers. For instance, products may contain a high level of fibre and protein content, added vitamins and other nutrients, such as calcium and iron, less sugar and additional multigrain ingredients. Positional claims are strategic in food marketing because, in many cases, food choices are made at the point of purchase [22]. Claims can help consumers identify breakfast cereals with more health benefits and, therefore, assist them in selecting a better diet. It is important to note that nutrition and health labelling is controlled at a European level by Regulation (EC) 1924/2006 and its subsequent amendments. This regulation defines a nutrition claim “as any claim which states, suggests or implies that a food has particular beneficial nutritional properties due to the presence, absence, increased or reduced levels of energy or of a particular nutrient or other substance” while defines a health claim as “any claim that states, suggests or implies that a relationship exists between a food category, a food or one of its constituents and health”. This study, following previous research, considers the two different types of claims in a single group.

Previous studies have observed the positive impact of health claims on consumers’ evaluations of food [23,24]. Moreover, as reported in previous research, breakfast cereals are one of the food categories that make a higher number of health and nutrition claims [25]. Hieke et al. [26] considered five European countries and reported that almost a third of cereal products in the market make nutritional claims. Later, Sussman et al. [25] revealed that, in the Australian market, 95% of the breakfast cereal products they surveyed made nutrition-, health- or other related claims.

Several studies have audited the prevalence of health claims in different EU and non-EU countries and analysed their impact on healthy diets [26,27]. Other studies have considered the impact of health claims on food choices [28,29]. The purpose of this paper is to focus on the introduction of new products in the breakfast cereals category, as well as examining the role of retailers and manufacturers in encouraging consumers’ purchase decisions with the release of new products making particular positional claims. The aim of this is to help the food industry to better understand the potential impact of their innovation strategies and package marketing information on the quality of consumer diets. In particular, the paper aims to answer the following questions:

- To what extent are UK food suppliers working to increase the availability of breakfast cereals with healthy and nutritious attributes?
- What are the companies that are leading the launching of these products? Which is the role of private labels?
- What claims are particularly important for the market success of newly developed breakfast cereals?

The results reveal that manufacturers and retailers are actively working on the introduction of new options with health and nutritional claims. Some of these claims do affect the success of the new product in the market, but no clear pattern can be identified.

The structure of the paper is as follows: it begins with the empirical section, which describes the data and methods. The following section presents a description of the introduction of new products in the UK breakfast cereals sector and the results of the survival analysis, considering the uptake of newly developed breakfast cereals. The final section provides the discussion and conclusions.

## 2. Materials and Methods

### 2.1. The Data

The first part of this study employs the Mintel Global New Products Database (GNPD) to present and provide an overview of the introduction of new breakfast cereals launched in the UK between January2000 and September 2019 (understanding it in a broad sense, not just considering new products, but also variations to existing products, and the relaunching and/or repackaging of products). The dataset contains information on 2262 new breakfast cereals launched in different types of retail stores between January 2000 and September 2019 by 282 manufacturing or retailing companies using 700 different brands.

**Figure A1:**
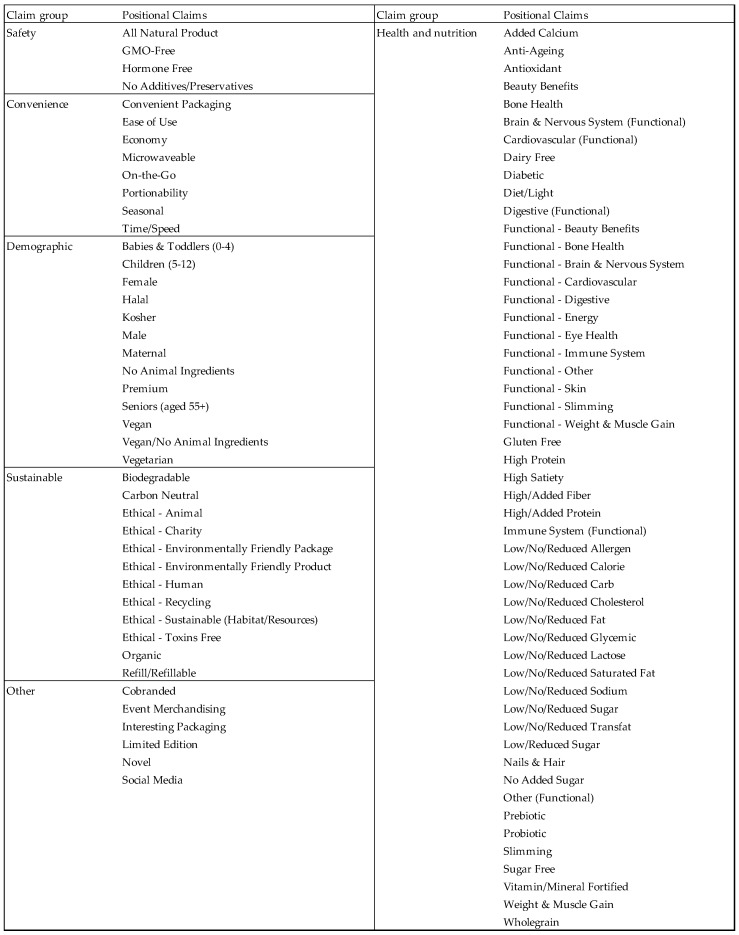
Claims considered under each claim category.

The products are classified into two categories, cold and hot cereals, and into 12 subcategories, as follows: corn cereals, granola, muesli, multigrain cereals, oat-based cereals, porridge, quinoa cereals, rice cereals, rye cereals, spelt cereals, wheat cereals and other. The database also contains information on the type of label (branded, private label), introduction price, and type of packaging. The fact that the dataset also provides information about the positioning claims of each product was particularly important for this study; 96 different claims were found in the dataset for breakfast cereals. This is important because such claims convey information to consumers about the product. For the analysis, these claims were classified into five groups: convenience (e.g., microwaveable), demographic (e.g., if designed for a particular demographic group), health and nutrition (e.g., low in calories), safety (e.g., no additives/preservatives), sustainable (e.g., organic) and others (e.g., limited edition, cobranded). See Figure A1 in Appendix A for more details on the particular claims considered under each claim category.

The second part of the study employs an assembled database combining data from GNPD and the Kantar Worldpanel Dataset (KWDS) for the UK. GNPD data were used to gather information on which new breakfast cereals were launched in the UK market in 2011. KWDS includes weekly records of all foods and beverages that were taken home from supermarkets and similar stores by UK households during the period 2013 to 2015. The breakfast cereals products observed in the GNPD database were identified in KWDS to follow its sales in UK retail and trace their durability in the market.

### 2.2. Analysis

In order to respond to the first and second research questions—exploring the trend in the introduction of breakfast cereals and identifying the leading suppliers—this research employs descriptive statistics (i.e., frequency distributions and cross-tabulations). To assess the influence of product claims on consumer acceptance, this study uses hazard-based duration models, which provide an explanation on the length of time that launched breakfast cereal products survived in the market. Duration models are based on the survivor function, which in our case is the probability of the product still being available in the market up to a specific time *t*, as follows:

(1) $$S_{\left(t\right)}=pPr\left\{T\geq t\right\}=1-F\left(t\right)=\int_{t}^{\infty }f\left(x\right)dx$$

where *T* is a continuous random variable, f(t) is its probability density function and F(t) is the cumulative distribution function. The distribution of *T* can also be expressed as the hazard function, which is the rate of occurrence of the event. In our case, the event means failing or disappearing from the market, and this is expressed as follows:

(2) $$h\left(t\right)=\underset{\Delta t\to \infty }{\lim}\frac{\mathrm{Pr}\left(t\leq T<t+\Delta t|T\geq t\right)}{\Delta t}$$

where the numerator is the conditional probability of occurrence during the period under consideration given that it has not occurred before, and the denominator is the length of the interval under consideration. The hazard function can also be expressed as follows:

(3) $$h\left(t\right)=\frac{f\left(f\right)}{S\left(t\right)}$$

In order to estimate the hazard function, different models can be used depending on the shape of the hazard and the features of the explanatory variables included in the model. A Cox’s proportional hazards model [30] was estimated for this research. Using the Cox model, specification of each individual follows its own survival function formulated as follows:

(4) $$h_{i}\left(t\right)=h\left(t;x_{i}\right)=h_{0}\left(t\right)exp\left(x_{i}^{’}\beta \right)$$

where $h_{0}\left(t\right)$ is a nonparametric function (baseline hazard at time *t*), $exp\left(x_{i}^{’}\beta \right)$ is a parametric function, and $x_{1},\ldots ,x_{k}$ are a pool of predictor variables. The dependent variable was the period (in years) that a product remained on the market. Table A1 in Appendix A presents the descriptive statistics for the variables considered for the econometric analysis.

## 3. Results

### 3.1. Overview of the Introduction of New Breakfast Cereals in the UK Market

**Figure 1:**
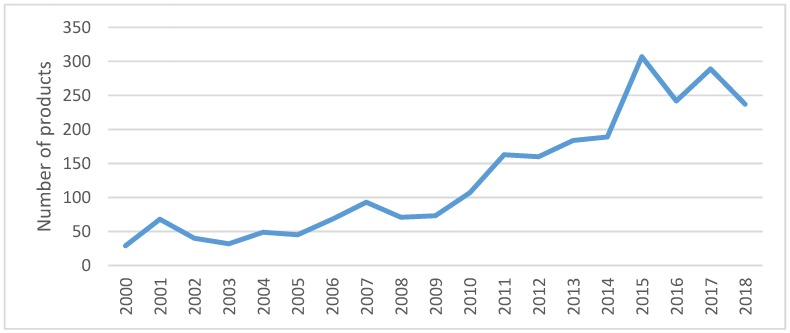
Number of breakfast cereals launched in the UK market 2000–2018. Source: Own elaboration based on Mintel’s Global New Products Database (GNPD) database.

Figure 1 below presents the evolution of new breakfast cereals introduced into the UK market between 2000 and 2018. A positive trend can be observed from 2000 to 2015, with a particularly large number of products marketed during the period 2011 to 2015. However, in 2016 and 2018, there is a clear drop in the number of new products.

**Figure 2:**
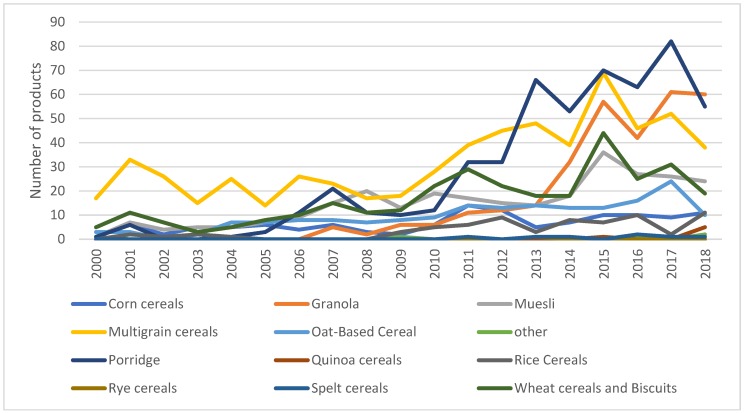
Subcategories of breakfast cereals launched in the UK market 2000–2018. Source: Own elaboration based on Mintel’s GNPD database.

Figure 2 disaggregates the information from Figure 1 by subcategories of breakfast cereals. It shows that, overall, multigrain breakfast cereals are the subcategory with the most launched products, comprising 25% of all breakfast cereals introduced during this period. However, since 2012, an increase in the introduction of porridge and granola products can also be observed. Porridge represents only 3% of the breakfast cereals introduced in 2000, while in the period 2012 to 2018, it represents between 20% and 35%.

**Figure 3:**
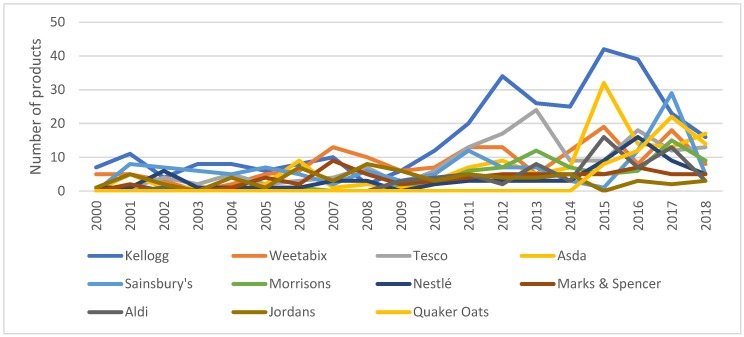
Companies launching breakfast cereals in the UK market 2000–2018. Source: Own elaboration based on Mintel’s GNPD database.

Due to the highly concentrated breakfast cereals market, companies invest in research and development in order to differentiate their products and increase their market share. Table 1 indicates that, for the period of January 2000 to September 2019, Kellogg’s was the top company for introducing products in the breakfast cereal category. The GNPD data also reveals that ten suppliers were responsible for the introduction of 50% of all the newly developed products during this period. Furthermore, six out of the ten top companies introducing new products are retailers and the new breakfast cereals introduced by retail firms account for 36% of the total. Figure 3 presents the number of products launched over time by the top 12 companies. Although Kellogg’s has always been a top developer, the period 2011 to 2016 was when it launched the largest number of products. We can also observe a considerable increase in the participation of retailers in new product development since 2011.

It is important to note that 96% of the newly launched products make a positional claim on the packaging. Table 2 lists the claims most frequently made by new breakfast cereals; the top ten claims account for more than 50% of the total claims made on packages during the period January 2000 to September 2019. The most frequent claim carried by newly launched breakfast cereals highlights that the new products are suitable for vegetarians, a demographic claim targeting a sector of consumers. Next, sustainability claims reporting the packaging to be environmentally friendly account for 8% of the total claims associated with new breakfast cereals. The third and fourth most frequent claims are health and nutrition claims, reporting the product to be wholegrain and with high or added fibre. These results are in line with previous reports [25] that the wholegrain claim is the most frequently made health claim on breakfast cereals.

**Figure 4:**
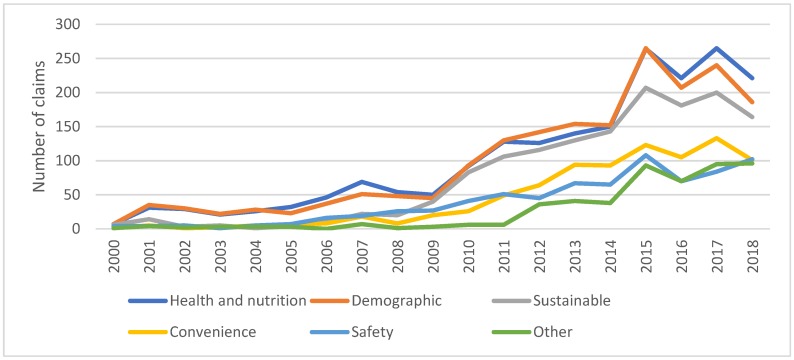
Subcategories of breakfast cereals launched in the UK market 2000–2018. Source: Own elaboration based on Mintel’s GNPD database.

Health and nutrition claims represent 27% of all the claims on the newly introduced breakfast cereals for the period January 2000 to September 2019. Figure 4 reveals that health and nutrition claims, together with demographics and sustainability claims, are the most important claims made by breakfast cereals over time. Moreover, in the period 2016 to 2018, health and nutritional claims are more frequent. It is worth noting that health and nutritional claims are often presented in combination with other claims (e.g., sustainability, safety, convenience or demographic claims); just 5% of the products make only health and nutritional claims. For 44% of the products, health and nutritional claims are combined with demographic and sustainability claims on the same package and 16% of the breakfast cereals make all these claims at once—health and nutritional, sustainability, safety, convenience and demographic claims. It is also important to mention that claims classified as safety in this study (i.e., all-natural product, genetically modified-free, hormone-free and no additives/preservatives) can also be considered to be health claims following the EU regulation 1924/2006.

The most frequent health claim for the period January 2000 to September 2019 is identifying the product as wholegrain. A total of 40% of the newly introduced products—1043 products—claim to be wholegrain. The claim emphasising a high presence of fibre is the second most frequent, with 37% of the newly launched products carrying this claim—970 new products. Products claiming to be fortified with vitamins and minerals and those declaring they have low or reduced levels of sodium comprise around 20%—549 and 491 products. Claims regarding allergens, such as low/no/reduced allergen or gluten-free are also important; these are made by 17% (446 products) and 16% (427 products) of the new products, respectively. Claims of containing less fat are carried by 12% of the newly developed breakfast cereals (303 products). Finally, the remaining health and nutritional claims are made by less than 10% of the new products. Table 3 shows the frequency of the appearance of health and nutritional claims made on breakfast cereals over time. It is important to note that those related to allergens have been increasing in recent years.

### 3.2. Effect of Nutrition and Health Claims on the Rate of Success of Newly Developed Breakfast Cereals in the UK

First, we undertook a descriptive analysis of the success of the new breakfast cereals (see Table 4, Table 5 and Table 6). Table 4 indicates an overall rate of success of 39.3% even though we can observe differences between the different product categories. The table also shows an index of success where the average rate of success is 100. New products associated with rice cereals, granola, muesli and oat-based cereals were the most successful categories (above the average rate of success).

Table 5 shows the degree of success by launch type. The greatest success is achieved in the relaunch of products, although there are only five of these. New products and new varieties or range extensions comprise the most launched products, and their degree of success is below the mean. It is also interesting that products that are relaunched with new packaging have a success rate of 50%. This could be related to a strategy of, after launching a new product, if it does not bring in the required sales (or, if it does, launching a new variety or range extensions), trying to apply new formulations, new packaging or relaunching the product. Even then, this does not ensure 100% success, however.

Table 6 presents the degree of success by claim on branded and private labels. On the branded products, the claims with the higher degree of success have a low glycaemic value (67%) and low transfats (33%). In the case of private-label products, the top claims are reduced allergens (80%) and reduced sugar (50%). In both cases—branded and private label—fibre is also an important claim. It is clear from the table that health claims are an important way to increase the product’s degree of success.

Second, we examined the factors influencing the success of the newly developed breakfast cereal products in the UK market using a duration model. Table 7 presents the estimated results for the Cox model considering the impact of the variables described in the annex (see Table A1). The goodness-of-fit results show that the model appropriately fits the data. We also implemented the proportional hazards assumption test, which indicated an absence of evidence contradicting the proportionality assumption.

The first variable considered in this analysis was the launch type (i.e., new formulation, new products, new variety or re-launch). The estimation revealed that the only launch strategy with a significant impact on the probability of the success of new breakfast cereals is the introduction of new packaging with a *p* < 0.10. The remaining launch strategies considered were not significant and were removed from the model. We can observe that the rate of failure of products launched with new packaging compared to other launch types (ceteris paribus) is 44% lower. The second and third variables considered were the type of company introducing the product (private company launching branded products or retailer launching private labels) and the introduction price. However, the *t* values were found to be non-significant for our model and they were, therefore, removed for the final estimation. Next, the origin of the product (UK or not) was considered, and interestingly this variable resulted in significant reduction of the probability of failure. However, its hazard ratio was minimal.

The impact of the type of breakfast cereal (i.e., granola, muesli, multigrain cereals, porridge) on the uptake of the product was also considered. The only category that was found to be significant was muesli, with a 76% lower chance of failure than other categories (ceteris paribus).

Considering the relevance of the ingredients listed on the product packaging (i.e., corn, wheat, oats, rice, rye, spelt and barley), only the wheat and rye results were significant and were, therefore, retained in the final model. We can observe from Table 7 that using rye as an ingredient in a newly developed breakfast cereal has a positive but minimal impact on its market success. On the other hand, including wheat as an ingredient has a negative effect on the uptake of this product. The estimated hazard of failure for breakfast cereals listing wheat as an ingredient is 3.4 times the hazard for other breakfast cereals without such a claim.

Regarding the utility of including claims, we can observe that the probability of failure for products not including claims is higher than for those making claims. In particular, not listing any claim on the cereal package increases the risk of failure 9.58 times compared to products that do include claims (ceteris paribus). When considering the different claims associated with the newly introduced breakfast cereals, only ten claims had a significant result and were, therefore, included in the final estimation. Half of the significant claims are health- and nutrition-related claims, three are sustainability claims, there is one safety claim and, finally, one convenience claim. The impact that the different claim groups have on the uptake of the products can be either positive or negative, depending on the claim. It is important to highlight that all claims with a positive impact appear to have a very small hazard ratio. This means that its effect is significant but very small; this is the case for products stating to have added calcium and making low/no/reduced glycaemic index health claims. Another claim with a small but positive impact on the success of new breakfast cereals is all-natural products, which we consider as a safety claim, but it can also have some health implications. Sustainability claims, such as ethical animal and organic, also increase the probability of success of new breakfast cereals. Other health and nutritional claims, such as improving the brain and nervous system, with low/no/reduced lactose and prebiotic, increase the probability of failure by 5.9, 8.2 and 10.5 times, respectively, compared with products not labelled with these claims. Finally, being an environmentally friendly product and being instant reduce the probability of success of breakfast cereals 3.174 and 3.969 times, respectively.

Interactions between subcategories and claims were considered in the analysis; the results were non-significant, and these variables were eliminated from the final estimation.

## 4. Discussion

The present study first explores whether food suppliers (i.e., manufacturers and retailers) are working to increase the range of breakfast cereals they offer and whether these new products are labelled with health and nutritional claims. Second, it identifies which suppliers are leading this process, and third it assesses to what extent health and nutrition claims made by breakfast cereals have an impact on their market success.

An increasing number of new breakfast cereals were introduced into the UK market during the period 2000 to 2015, with a clear reduction in the number of new products from 2016 to 2018. Our results show that 96% of the newly developed breakfast cereals launched from 2000 to 2019 do include positional claims. Therefore, we can state that manufacturers and retailers include health and other information to differentiate their products effectively and, as indicated by previous research [31], potentially help consumers to make informed food purchases.

Among the positional claims, we identified health and nutrition, sustainability, convenience and demographic attributes. The most common are demographic claims, which are included in 77% of all new breakfast cereals. It is essential to note that being suitable for vegetarians comprises 66% of the products with demographic claims. Moreover, if we also include products informing customers about the absence of animal ingredients or reporting to be suitable for vegans, this encompasses 97% of all the new products making demographic claims. Taking into account that a reduction in the use of animal ingredients is an important aspect of achieving a rich and healthy diet, we can state that, by including these claims, suppliers are, in a way, assisting consumers to not only make ethical and sustainable choices but also healthier ones.

The second most numerous claims are health- and nutrition-related, present in 27% of the new breakfast cereals. Although we identified 51 different claims for this group, the most frequent are those reporting that the product is wholegrain, which appears in 40% of the products making health claims. Informing customers about the high level of fibre in the new product is also a very common claim. These two claims are particularly important in improving the nutrition levels of Western diets and they can explain the higher consumption of high-fibre cereals per person compared to other breakfast cereals reported in the introduction section. Health claims reporting the absence of allergens have also become increasingly frequent since 2014. These claims can have a positive impact on the section of the population suffering from food allergies and food intolerances that require them to avoid specific ingredients in their diets. Adverse reactions to foods have been reported to represent a key health-issue in Western societies [32]. It is also important to note that safety claims (i.e., all-natural products, genetically modified-free, hormone free and no additives/preservatives) are made by 32% of the newly introduced breakfast cereals and these claims also have health and nutritional implications.

Our results demonstrate that the breakfast cereal industry in the UK follows the diversification model defined by [19]. A small group of leading suppliers, such as Kellogg’s, Weetabix, Nestle and the big retailers (i.e., Sainsbury’s, Tesco, Asda, etc.) are directing the research and development in the breakfast cereals sector. The same companies leading research and development are, to a large extent, the ones leading market sales in the breakfast cereals market. This is an important point to consider when developing policies to improve diets. Consumers purchase the options made available to them by retailers and manufacturers [21]. In line with [31,33,34], our results suggest that the research and development departments of manufacturers, and especially, retail companies, can help consumers move towards healthier diets by deciding what to place on the shelves. During the last ten years, companies have been focusing on the development and commercialisation of multigrain breakfast cereals, porridge and granola. The introduction of wheat and corn cereals has been reducing, and it appears to be following a decreasing trend. One of the constraints upon the rich and healthy diet noted as necessary by [1] is the limited consumption of wholegrain and assorted cereals, seeds and fruits. Expansion of the number of multigrain cereals, porridge and granola on offer can help to improve the limitations noted above. Therefore, it can be suggested that companies are working towards an improvement in the availability of healthy and nutritious breakfast options in the UK. However, not all new products are accepted by consumers, and other elements, such as sociodemographic characteristics, education, social pressure, price, marketing and communication of product benefits, will determine the uptake of the new offerings and the consequent impact on consumers’ diets [29]. Collaboration between food suppliers, policymakers and health organisations is, therefore, needed to inform citizens regarding healthy diets and to promote healthy food. Consumer knowledge, attitude and purchase intention also shape manufacturers and retailers’ research and development decisions.

In line with previous research on the success of newly developed products, our results support the suggestion that a limited proportion of newly launched products succeed in the market in the mid- to long-term. We found that less than 40% of the newly introduced breakfast cereals succeeded in the UK market in the medium–long term. This result highlights the complexity of finding a good new product development strategy and explains why this activity is mainly concentrated among big companies that can afford the economic implications of failing in the food market, and who can also undertake broader market research strategies to capture consumers’ needs.

Regarding the impact of health and nutritional claims on the success of newly developed breakfast cereals for the UK market, there is no clear pattern. Our Cox regression results clearly show that placing claims informing consumers about the benefits of the newly developed breakfast cereals on the packaging does positively influence the success of these products in the market. That is, when consumers are informed about what they can get from a newly developed product in comparison to other options, they are more likely to purchase it. This result is in line with [28]. The estimation shows that some health and nutritional claims have a positive effect on the success of new breakfast cereals, while others have a negative impact. The impact of nutritional claims on the success of newly developed breakfast cereals is claim-specific. Moreover, for our sample, all claims with a positive impact appear to have a very small hazard ratio. This may be due to the sample size and the number of years being considered. The health claims found to have a positive effect on the uptake of the new products are added calcium, low/no/reduced glycaemic index, all-natural product, ethical animal and organic. On the other hand, our results show that claims such as improving the brain and nervous system, low/no/reduced lactose, prebiotic, environmentally friendly product and instant increase the probability of failure of newly developed breakfast cereals in the UK market.

We also found that the ingredients listed on the product packaging have an impact on the uptake of the products. Our results show that using wheat as an ingredient reduces the level of success of the newly developed breakfast cereals, while including rye as an ingredient increases its success. This result may be related to the increase in the incidence of celiac disease and the negative information reported on social media about gluten and wheat as a cause of obesity and other health problems [35]. Our results show that newly launched muesli products are more accepted by consumers than other categories of breakfast cereals. Considering that the composition of muesli is grains, fresh or dried fruits, seeds and nuts, it seems that consumers welcome healthy breakfast cereal options. Finally, informing consumers about the UK origin of the cereals was identified as having a positive impact on the purchase of the product.

Interactions between subcategories and claims were considered in the analysis, but the results were non-significant and were, therefore, eliminated from the final estimation. The lack of significance found in these interactions may be due to the sample size. The products introduced over one year might offer limited variability within the different categories and claims.

Taking these results together, we can suggest that manufacturers and retailers are assuming a proactive and important role in research and development to capture consumers’ needs and, therefore, help consumers to move towards healthier and better diets. Positional claims carried by food products need to be very clear on the potential benefits and risks of the products in order to be of real help to consumers in their food-purchasing decisions. Future research can consider a longer time period and the nutritional value associated with the newly introduced products.

## Figures and Tables

**Table 1:** Frequency of new products released by companies January 2000–September 2019.

Company	Frequency	Percent
Kellogg’s	332	14.68
Weetabix	164	7.25
Tesco	161	7.12
Asda	145	6.41
Sainsbury’s	135	5.97
Morrisons	81	3.58
Nestlé	75	3.32
Marks and Spencer	74	3.27
Aldi	73	3.23
Jordans	69	3.05
Rest of Companies	63	51.15

Source: Own elaboration based on Mintel’s GNPD database.

**Table 2:** Frequency of product claims January 2000–September 2019.

Claims	Frequency	Percent
Vegetarian	1727	10.80
Ethical—Environmentally Friendly Package	1345	8.41
Wholegrain	1043	6.52
High/Added Fibre	970	6.07
No Additives/Preservatives	716	4.48
Ethical—Recycling	712	4.45
Vitamin/Mineral Fortified	549	3.43
Low/No/Reduced Sodium	491	3.07
Social Media	468	2.93
Low/No/Reduced Allergen	446	2.79
Gluten Free	427	2.67
Microwaveable	400	2.50
Ease of Use	361	2.26
Time/Speed	348	2.18
Vegan	304	1.90
Low/No/Reduced Fat	303	1.90
Rest of Claims	5379	33.64

Source: Own elaboration based on Mintel’s GNPD database.

**Table 3:** Product frequency of health and nutrition claims by year from 2004 to 2018.

	2004	2005	2006	2007	2008	2009	2010	2011	2012	2013	2014	2015	2016	2017	2018
Wholegrain	4	5	23	29	26	25	53	71	62	71	80	151	111	152	96
High/Added Fibre	10	7	21	20	18	2	22	71	66	65	75	132	116	128	105
No Additives/Preservatives	3	6	15	16	25	23	37	46	42	58	54	96	62	77	81
Vitamin/Mineral Fortified	9	14	13	17	13	13	25	33	50	33	30	70	47	58	49
Low/No/Reduced Sodium	3	13	6	23	6	18	33	40	36	33	33	52	54	72	33
Low/No/Reduced Allergen	2	2	2	10	4	12	15	15	18	18	26	51	60	63	89
Gluten Free	1	1	1	9	4	7	13	12	15	15	21	50	63	68	88
Low/No/Reduced Fat	7	11	11	20	11	10	23	23	16	24	21	33	17	29	11
Functional—Cardiovascular	0	0	0	0	0	0	0	0	0	0	0	31	45	58	32
Low/No/Reduced Sugar	1	13	7	16	11	12	19	19	16	17	17	8	0	0	0
Low/No/Reduced Transfat	0	0	0	1	3	4	5	11	14	9	8	33	20	16	16
No Added Sugar	0	0	0	0	0	0	0	0	0	0	0	17	28	27	39
Bone Health	0	0	0	0	0	1	1	4	3	4	13	32	22	20	11
Functional—Energy	0	0	0	0	0	0	0	0	0	0	0	28	31	27	31
Low/No/Reduced Saturated Fat	0	1	5	6	4	5	7	10	7	5	12	9	15	11	9
Cardiovascular (Functional)	0	1	1	4	2	3	14	20	16	15	25	4	0	0	0
Functional—Bone Health	0	0	0	0	0	0	0	0	0	0	0	24	22	20	11
High/Added Protein	0	0	0	0	0	0	0	0	0	0	0	13	21	26	16
Dairy Free	0	0	0	0	0	0	0	0	0	0	0	9	10	28	23
Low/Reduced Sugar	0	0	0	0	0	0	0	0	0	0	0	10	13	16	15
Functional—Other	0	0	0	0	0	0	0	0	0	0	0	14	12	15	13
Rest of Health Claims	11	9	15	17	21	22	31	47	38	49	53	88	87	87	107

**Table 4:** Degree of success of new breakfast cereals by product category.

Categories	Fully	Partial	Success	Total	Percentages				Success
	Failed	Success			Failed	Partial	Success	Total	Index
Rice Cereals	0	1	5	6	0.0	16.7	83.3	100.0	2.12
Granola	4	1	6	11	36.4	9.1	54.5	100.0	1.39
Muesli	6	2	9	17	35.3	11.8	52.9	100.0	1.35
Oat-Based Cereal	5	5	7	17	29.4	29.4	41.2	100.0	1.05
Porridge	9	9	11	29	31.0	31.0	37.9	100.0	0.97
Corn Cereals	4	5	5	14	28.6	35.7	35.7	100.0	0.91
Wheat Cereals	10	7	9	26	38.5	26.9	34.6	100.0	0.88
Multigrain Cereals	16	11	12	39	41.0	28.2	30.8	100.0	0.78
Spelt Cereals	1	0	0	1	100.0	0.0	0.0	100.0	0.00
Wheat Biscuits	3	0	0	3	100.0	0.0	0.0	100.0	0.00
Total	58	41	64	163	35.6	25.2	39.3	100.0	1.00

Source: Own elaboration based on GNPD and Kantar Worldpanel Dataset (KWDS) database.

**Table 5:** Degree of success by launch type.

	Fully	Partial	Success	Total	Percentages				Success
	Failed	Success			Failed	Partial	Success	Total	Index
New Formulation	5	3	6	14	35.7	21.4	42.9	100.0	1.09
New Packaging	7	11	18	36	19.4	30.6	50.0	100.0	1.27
New Variety/Range Extension	21	16	22	59	35.6	27.1	37.3	100.0	0.95
Relaunch	2	0	3	5	40.0	0.0	60.0	100.0	1.53
New Product	23	11	15	49	46.9	22.4	30.6	100.0	0.78
Total	58	41	64	163	35.6	25.2	39.3	100.0	1.00

Source: Own elaboration based on GNPD and KWDS database.

**Table 6:** Degree of success of breakfast cereals by claim on branded and private labels.

Claim	Fully	Partial	Success	Total	Percentages	Partial	Success	Total
	Failed	Success			Failed			
**Branded**								
High/Added Fibre	11	7.00	23	41	26.80	17.1	56	100
Low/No/Reduced Allergen	3	3.00	4	10	30.00	30	40	100
Low/No/Reduced Fat	6	1.00	5	12	50.00	8.3	41.7	100
Low/No/Reduced Glycaemic	1	0.00	2	3	33.30	0	66.7	100
Low/No/Reduced Lactose	1	2.00	0	3	33.30	66.7	0	100
Low/No/Reduced Saturated Fat	2	2.00	3	7	28.60	28.6	42.9	100
Low/No/Reduced Sodium	10	7.00	16	33	30.30	21.2	48.5	100
Low/No/Reduced Sugar	6	2.00	9	17	35.30	11.8	52.9	100
Low/No/Reduced Transfat	0	1.00	2	3	0.00	33.3	66.7	100
**Private Label**								
High/Added Fibre	10	9.00	11	30	33.30	30	36.7	100
Low/No/Reduced Allergen	0	1.00	4	5	0.00	20	80	100
Low/No/Reduced Fat	5	4.00	2	11	45.50	36.4	18.2	100
Low/No/Reduced Saturated Fat	2	0.00	1	3	66.70	0	33	100
Low/No/Reduced Sodium	4	3.00	0	7	57.10	42.9	0	100
Low/No/Reduced Sugar	1	0.00	1	2	50.00	0	50	100
Low/No/Reduced Transfat	3	4.00	1	8	37.50	50	12.5	100
**Total**								
High/Added Fibre	21	16.00	34	71	29.60	22.5	47.9	100
Low/No/Reduced Allergen	3	4.00	8	15	20.00	26.7	53.3	100
Low/No/Reduced Fat	11	5.00	7	23	47.80	21.7	30.4	100
Low/No/Reduced Glycaemic	1	0.00	2	3	33.30	0	66.7	100
Low/No/Reduced Lactose	1	2.00	0	3	33.30	66.7	0	100
Low/No/Reduced Saturated Fat	4	2.00	4	10	40.00	20	40	100
Low/No/Reduced Sodium	14	10.00	16	40	35.00	25	40	100
Low/No/Reduced Sugar	7	2.00	10	19	36.80	10.5	52.6	100
Low/No/Reduced Transfat	3	5.00	3	11	27.30	45.5	27.3	100.00

Source: Own elaboration based on GNPD and KWDS database.

**Table 7:** Cox regression time-constant variables label.

	Rate of Failure					
	Coeff.	St. Err.	Haz. Rat.	St. Err.	*t* Ratio	Sig.
New Packaging	−0.579	0.334	0.561	0.187	−1.730	*
No claim	2.260	0.660	9.585	6.324	3.430	***
Muesli	−1.447	0.661	0.235	0.156	−2.190	**
Has wheat as an ingredient	1.211	0.333	3.358	1.119	3.640	***
Has rye as an ingredient	−38.342	0.956	8.20 × 10^−18^	7.84 × 10^−18^	−40.100	***
Dummy added calcium	−38.693	1.054	5.78 × 10^−18^	6.09 × 10^−18^	−36.710	***
Dummy all-natural product	−38.736	1.019	5.53 × 10^−18^	5.64 × 10^−18^	−38.020	***
Dummy brain nervous system	1.773	0.328	5.890	1.933	5.400	***
Dummy ethical animal	−38.457	1.213	7.32 × 10^−18^	8.87 × 10^−18^	−31.710	***
Dummy environmentally friendly product	1.378	0.419	3.969	1.664	3.290	***
Dummy low/no/reduced glycaemic	−40.508	0.861	9.41 × 10^−19^	8.09 × 10^−19^	−47.070	***
Dummy low/no/reduced lactose	2.100	0.420	8.166	3.432	5.000	***
Dummy organic	−39.196	0.817	3.49 × 10^−18^	2.85 × 10^−18^	−47.970	***
Dummy prebiotic	2.352	0.412	10.508	4.334	5.700	***
Dummy instant	1.155	0.653	3.174	2.073	1.770	*
Dummy UK ingredients	−38.114	1.085	1.03 × 10^−17^	1.12 × 10^−17^	−35.120	***
Log likelihood ratio test	−230.54					
Wald chi2 (15)	12710.55	***				
Number of observations	123					

*p* < 0.10 * *p* < 0.05 ** *p* < 0.01 ***. Coeff. (Coefficient); St. Err. (Standard Error); Haz. Rat. (Hazard Ratio) and Sig. (Significance).

## Appendix A

**Table A1:** Descriptive statistics.

Variable	Obs.	Mean	St. Dev.	Min	Max
Number of years a product has been sold	152	3.00	2.05	0	5
New Formulation	152	0.28	0.45	1	1
New Packaging	152	0.24	0.43	2	2
New Product	152	0.38	0.49	3	3
New variety	152	0.07	0.26	4	4
Product category (dummies)					
Multigrain cereals	152	0.24	0.43	0	1
Porridge	152	0.06	0.24	0	1
Granola	152	0.18	0.38	0	1
Muesli	152	0.09	0.16	0	1
Ingredients (dummies)					
Wheat	152	0.55	0.50	0	1
Corn	152	0.18	0.38	0	1
Oats	152	0.64	0.48	0	1
Rice	152	0.33	0.47	0	1
Rye	152	0.02	0.14	0	1
Spelt	152	0.06	0.24	0	1
Barley	152	0.39	0.49	0	1
Quinoa	152	0.00	0.00	0	1
Dummy branded (0) and private label (1)	152	0.40	0.49	0	1
Dummy 1 UK product or UK ingredients	152	0.01	0.11	0	1
Type of claim (dummies)					
Added Calcium	152	0.01	0.08	0	1
All Natural Product	152	0.03	0.16	0	1
Anti-Ageing	152	0.00	0.00	0	0
Antioxidant	152	0.02	0.14	0	1
Beauty Benefits	152	0.01	0.08	0	1
Bone Health	152	0.03	0.16	0	1
Brain & Nervous System (Functional)	152	0.01	0.11	0	1
Carbon Neutral	152	0.00	0.00	0	0
Cardiovascular (Functional)	152	0.13	0.34	0	1
Children (5–12)	152	0.09	0.29	0	1
Cobranded	152	0.01	0.08	0	1
Convenient Packaging	152	0.04	0.20	0	1
Digestive (Functional)	152	0.06	0.24	0	1
Ease of Use	152	0.07	0.26	0	1
Economy	152	0.07	0.25	0	1
Ethical—Animal	152	0.01	0.11	0	1
Ethical—Charity	152	0.05	0.22	0	1
Ethical—Environmentally Friendly Package	152	0.61	0.49	0	1
Ethical—Environmentally Friendly Product	152	0.07	0.26	0	1
Ethical—Human	152	0.00	0.00	0	0
Event Merchandising	152	0.00	0.00	0	0
Female	152	0.00	0.00	0	0
Gluten-Free	152	0.08	0.27	0	1
GMO-Free	152	0.05	0.22	0	1
Halal	152	0.08	0.27	0	1
High Protein	152	0.00	0.00	0	0
High Satiety	152	0.03	0.16	0	1
High/Added Fiber	152	0.46	0.50	0	1
Immune System (Functional)	152	0.03	0.18	0	1
Interesting Packaging	152	0.00	0.00	0	0
Kosher	152	0.13	0.33	0	1
Limited Edition	152	0.03	0.18	0	1
Low/No/Reduced Allergen	152	0.09	0.29	0	1
Low/No/Reduced Calorie	152	0.00	0.00	0	0
Low/No/Reduced Carb	152	0.00	0.00	0	0
Low/No/Reduced Cholesterol	152	0.00	0.00	0	0
Low/No/Reduced Fat	152	0.14	0.35	0	1
Low/No/Reduced Glycemic	152	0.01	0.11	0	1
Low/No/Reduced Lactose	152	0.01	0.11	0	1
Low/No/Reduced Saturated Fat	152	0.06	0.24	0	1
Low/No/Reduced Sodium	152	0.25	0.43	0	1
Low/No/Reduced Sugar	152	0.11	0.31	0	1
Low/No/Reduced Transfat	152	0.07	0.26	0	1
Male	152	0.00	0.00	0	0
Microwaveable	152	0.20	0.40	0	1
No Additives/Preservatives	152	0.28	0.45	0	1
No Animal Ingredients	152	0.07	0.25	0	1
Novel	152	0.00	0.00	0	0
On-the-Go	152	0.01	0.11	0	1
Organic	152	0.06	0.24	0	1
Other (Functional)	152	0.06	0.24	0	1
Portionability	152	0.00	0.00	0	0
Prebiotic	152	0.01	0.11	0	1
Premium	152	0.04	0.20	0	1
Seasonal	152	0.00	0.00	0	0
Seniors (aged 55+)	152	0.00	0.00	0	0
Slimming	152	0.01	0.11	0	1
Social Media	152	0.00	0.00	0	0
Time/Speed	152	0.12	0.32	0	1
Vegan	152	0.07	0.25	0	1
Vegetarian	152	0.76	0.43	0	1
Vitamin/Mineral Fortified	152	0.22	0.41	0	1
Weight & Muscle Gain	152	0.00	0.00	0	0
Wholegrain	152	0.47	0.50	0	1
